# Laboratory-Induced Bifenthrin, Flonicamid, and Thiamethoxam Resistance and Fitness Costs in *Rhopalosiphum padi*

**DOI:** 10.3390/toxics11100806

**Published:** 2023-09-24

**Authors:** Hina Gul, Ihsan ul Haq, Ali Güncan, Farman Ullah, Nicolas Desneux, Xiaoxia Liu

**Affiliations:** 1 MARA Key Laboratory of Pest Monitoring and Green Management, Department of Entomology, College of Plant Protection, China Agricultural University, Beijing 100193, China; gulhina@cau.edu.cn; 2 Insect Pest Management Program, Institute of Plant and Environmental Protection, National Agricultural Research Centre, Islamabad 44000, Pakistan; 3 Department of Plant Protection, Faculty of Agriculture, Ordu University, 52200 Ordu, Türkiye; guncan.ali@gmail.com; 4 State Key Laboratory for Managing Biotic and Chemical Threats to the Quality and Safety of Agro-Products, Institute of Plant Protection and Microbiology, Zhejiang Academy of Agricultural Sciences, Hangzhou 310021, China; farmanullah787@gmail.com; 5 INRAE, Université Côte d’Azur, CNRS, UMR ISA, 06000 Nice, France

**Keywords:** resistance selection, demographic parameters, wheat aphids, chemical application, life table

## Abstract

The bird cherry-oat aphid, *Rhopalosiphum padi* (L.) (Hemiptera: Aphididae) is one of the most economically important pests of wheat crops worldwide. Thiamethoxam, bifenthrin, and flonicamid are extensively used insecticides for controlling this key pest. However, the indiscriminate use of chemical insecticides has led to the development of resistance in insects. In this study, we assessed the development of selection-induced resistance to bifenthrin, flonicamid, and thiamethoxam under controlled laboratory conditions. Additionally, we employed the age-stage, two-sex life table method to examine the fitness of *R. padi*. After ten generations of selection, bifenthrin-, flonicamid-, and thiamethoxam-resistant strains of *R. padi* were developed with resistance levels of 34.46, 31.97, and 26.46-fold, respectively. The life table analysis revealed a significant decrease in adult longevity and fecundity in these resistant strains compared to susceptible strain. Furthermore, the key demographic parameters such as net reproductive rate (*R*_0_) and reproductive days exhibited a significant reduction in all resistant strains, while the intrinsic rate of increase (*r*) and finite rate of increase (*λ*) were decreased only in resistant strains to bifenthrin and thiamethoxam. Taken together, these findings provide a comprehensive understanding of laboratory-induced insecticide resistance evolution and the associated fitness costs in *R. padi*. This knowledge could help to design resistance management strategies against this particular pest of wheat.

## 1. Introduction

The bird cherry-oat aphid, *Rhopalosiphum padi* (Hemiptera: Aphididae) is a significant global threat to wheat due to its economic impact. Its damage to wheat is characterized through direct feeding and transmission of various plant viruses [1]. The damage rate of this key pest is increasing yearly in China’s wheat-growing regions, particularly in the southern parts. While several methods have been developed and used to control major crop pests [2,3,4,5,6], chemical applications remain a viable approach for pests. The frequent and indiscriminate use of insecticides has led to the development of resistance in *R. padi* against several classes of insecticides including pyrethroids, neonicotinoids, and organophosphates [7,8]. The failure of chemically based insect pest management is thought to be mainly due to the selection of pesticide resistance [9,10,11,12]. Furthermore, the widespread chemical applications in the field causes the non-target effects on beneficial insects, endangering human health, and posing risks to the environment [13].

Compared to other individuals of the same species, an insect’s fitness is determined by its capacity to endure and reproduce within a specific environment. When alleles confer better fitness in one environment, such as in the presence of insecticide selection pressure, but result in reduced fitness in a different environment, such as when insecticides are not present, this trade-off between multiple attributes is known as a fitness cost [14]. Insects bearing resistance strains experience these fitness costs that impact their reproductive and survival capabilities [10,11,15]. Additionally, in specific field conditions, these fitness costs may prevent the emergence of resistance within a population [16]. Insect populations that have developed insecticide resistance often exhibited reduced fitness due to the significant energetic cost associated with evolutionary processes [17]. Various studies have found that fitness costs influence insecticide resistance in a variety of insect pests, including *Aphis gossypii* Glover (Hemiptera: Aphididae), *Plutella xylostella* [18] (Lepidoptera: Plutellidae), *Nilaparvata lugens* (Stål) (Hemiptera: Delphacidae), *Thrips hawaiiensis* (Morgan) (Thysanoptera: Thripidae) and *Musca domestica* L. (Diptera: Muscidae) [19,20,21,22,23]. The sulfoxaflor-resistant *A. gossypii* exhibited a reduction in relative fitness cost, with a value 0.917 representing the ratio of the net reproductive rate of resistant strain to that of susceptible strain [24]. Similarly, the reduction in relative fitness was calculated as 0.83 in sulfoxaflor-resistant *Myzus persicae* (Sulzer) (Hemiptera: Aphididae) when compared to susceptible insects [25]. Several insect pests have demonstrated resistance to insecticides coupled with associated fitness costs [10,11,23,24,26]. The laboratory induced resistance development of bifenthrin, flonicamid, and thiamethoxam and the related fitness costs have not yet been studied in *R. padi*. Investigating the selection-induced resistance development in the target pest is crucial for assessing the risks associated with the indiscriminate use of insecticides.

The life table analysis in demographic toxicology is an effective approach for determining how various environmental factors, such as temperatures or insecticides, and secondary plant metabolites impact the fecundity, reproduction, life expectancy, growth, and survival of insect populations [18]. Traditional life tables were only based on the female population, which omitted the male population, and excluded information on individual variations and developmental stages [27]. Consequently, using age-stage, two-sex life tables minimized these disadvantages of female-based life tables by incorporating data from both sexes of a population [18].

Therefore, one of the the objectives of this study was to reveal the development of bifenthrin, flonicamid, and thiamethoxam resistance in *R. padi* over ten generations of continuous selection pressure. Additionally, we assessed the overall fitness of the bifenthrin-, flonicamid-, and thiamethoxam-resistant strains in comparison to susceptible aphids using the age-stage, two-sex life table method. The findings concerning selection-induced resistance and the associated fitness costs of these insecticides hold the potential to provide valuable insights into steps of resistance evolution within *R. padi* populations. 

## 2. Materials and Methods

### 2.1. Insect Rearing and Insecticides

The laboratory strain of *R. padi* was originally collected from a wheat field and reared for several years at the National Agricultural Research Centre, Islamabad, Pakistan, without any exposure to chemical insecticides. The colony was maintained on insecticide-free wheat seedlings under standard laboratory conditions, with a temperature of 18 °C, relative humidity (RH) of 60 ± 5%, and a photoperiod of 16:8 light:dark. The commercial insecticides used in this study, i.e., bifenthrin (Picador^®^ 10% EC), flonicamid (Super^®^ 75% WDG), and thiamethoxam (Actara^®^ 25 WG) were provided by Syngenta Pakistan Ltd. (Karachi, Pakistan) and Star Industries (pvt) Ltd. (Karachi, Pakistan).

### 2.2. Laboratory Bioassays

To assess the dose-response toxicity of bifenthrin, flonicamid, thiamethoxam insecticides on *R. padi*, we prepared test concentrations of each insecticide from their respective stock solutions. These tested concentrations, which include bifenthrin at 80, 40, 20, 10, and 5 ppm, flonicamid at 20, 10, 5, 2.5, and 1.25 ppm, and thiamethoxam at 40, 20, 10, 5, and 2.5 ppm, were determined following preliminary bioassays conducted on the laboratory strain of *R. padi*. These serial concentrations of all insecticides were applied separately to both the adaxial and abaxial leaf surfaces of wheat plants (five-leaf stage) until runoff. Distilled water was used as a control. The treated plants were left to air-dry at room temperature. Each insecticide concentration was replicated three times. In each replicate, thirty adult apterous parthenogenetic individuals were used. The treated wheat plants, with aphids, were set up in laboratory conditions (maintained at 18 °C, 60 ± 5% RH, and a photoperiod of 16:8 light:dark). Mortality was checked after 48 h of exposure. Aphids were considered dead if they displayed no movement upon gentle touch. Toxicity assessment was evaluated using the software POLO Plus 2.0 (LeOra Software Inc., Berkeley, CA, USA).

### 2.3. Establishment of Resistant Strain Populations

The resistant strains of bifenthrin, flonicamid, and thiamethoxam were developed from a susceptible population of *R. padi* through continuous selection pressure over ten generations. The acute toxicity of bifenthrin, flonicamid, and thiamethoxam was evaluated for each generation. The results of the parent aphid bioassays indicated a gradual increase in all insecticide concentrations throughout the selection experiment. In each generation of *R. padi*, the resistance ratio (RR) was calculated by dividing the LC_50_ values of the resistant strains by the LC_50_ values of the susceptible strains. The susceptible strain of *R. padi* was maintained using fresh insecticide-free wheat plants. Each strain was separately kept under controlled laboratory conditions (maintained at 18 °C, 60 ± 5% RH, and a photoperiod of 16:8 light:dark).

### 2.4. Evaluating the Fitness Cost

The fitness of bifenthrin-, flonicamid-, and thiamethoxam-resistant strains of *R. padi* compared to the susceptible strains was assessed using an age-stage, two-sex life table analysis. For each strain, we collected 500 apterous adults of *R. padi* and transferred to wheat seedlings without the application of insecticides. After a 24 h period, we individually placed 40 newly born nymphs of *R. padi* from susceptible and bifenthrin-, flonicamid-, and thiamethoxam-resistant strains into micro cages containing insecticide-free fresh wheat plant leaves. Each aphid was considered a single replicate for all *R. padi* strains. The nymphs of susceptible and all resistant strains were examined daily, and data related to fecundity, longevity, developmental time, and mortality were recorded. Newly born nymphs were counted and removed every day during the reproductive stages. All experiments were carried out under laboratory conditions at 18 °C with relative humidity (RH) 60 ± 5%, and a photoperiod of 16:8 light:dark.

### 2.5. Life Table Data Analysis

The age-stage, two-sex life table method [28,29] was used to examine the life table data of susceptible and bifenthrin-, flonicamid-, and thiamethoxam-resistant strains of *R. padi*. The TWOSEX-MSChart computer program [30,31] was used to assess the life history traits, including developmental stages, fecundity (*F*), adult longevity, adult pre-reproductive period (APRP), and total pre-reproductive period (TPRP), reproductive days (*RP_d_*) as well as key demographic parameters such as the net reproductive rate (*R*_0_), the intrinsic rate of increase (*r*), the finite rate of increase (*λ*), and the mean generation time (*T*) [28,30].

The age-stage survival rate *s_xj_* was calculated as;

(1) $$s_{xj}=\frac{n_{xj}}{n_{01}}\;$$

where *n*_01_ is the number of insects used at the beginning of the life table study and *n_xj_* is the number of insects surviving to age *x* and stage *j* [29]. 

The age-specific survival rate (*l_x_*) and age-specific fecundity (*m_x_*) were calculated as:

(2) $$l_{x}=\sum_{j=1}^{\beta }s_{xj}$$

(3) $$m_{x}=\frac{\sum_{j=1}^{\beta }s_{xj}f_{xj}}{\sum_{j=1}^{\beta }s_{xj}}$$

where *s_xj_* represents the possibility that a newly born nymph will survive to age *x* and stage *j*. *β* shows number of stages, while *f_xj_* represents age-stage specific fecundity of the individual at age *x* and stage *j*.

The *RP_d_* shows days of reproduction and was estimated using Equation (4):

(4) $$RP_{d}=\frac{\sum_{x=1}^{N_{f}}D_{x}}{N_{f}}$$

where *N_f_* represents the number of female adults and *D_x_* shows the number of days that a female produced offspring [32].

The *r* shows the intrinsic rate of increase when the time reach infinity, and the population achieve stable age-stage distribution. The insect’s population might be increased at a rate per unit of time. The *r* was calculated using the interactive bisection method and corrected with the Euler–Lotka equation with age indexed from 0 according to [33]:

(5) $$\sum_{x=0}^{\infty }e^{-r\left(x+1\right)}l_{x}m_{x}=1$$

The *λ* represents the finite rate of increase when time reaches infinity and population attains a stable age-stage distribution. The population size will increase at the rate of *λ* per time unit. The *λ* was estimated using Equation (6):

(6) $$\lambda =e^{r}$$

The *R*_0_ indicates the total number of nymphs laid by a single female until death. The *R*_0_ was calculated using Equation (7):

(7) $$R_{0}=\sum_{x=0}^{\infty }l_{x}m_{x}$$

The *T* represents the time required for a population to increase to *R*_0_-fold its current size at a stable rate of increase. The *T* was calculated using Equation (8):

(8) $$T=\frac{\ln R_{0}}{r}$$

The *e_xj_* shows the predicted duration that an individual of age *x* and stage *j* will survive. The *e_xj_* was calculated according to Chi and Su [34] using Equation (9):

(9) $$e_{xj}=\sum_{i=x}^{\infty }\sum_{y=j}^{\beta }{s^{\prime }}_{iy}$$

where *s′_iy_* indicates the probability that an individual aphid of age *x* and stage *j* will survive to age *i* and stage *y* by assuming s′ = 1.

*v_xj_* shows the dedication to future offspring at age *x* and stage *j*. The *v_xj_* was calculated using Equation (10) according to [35]

(10) $$v_{xj}=\frac{e^{r\left(x+1\right)}}{s_{xj}}\sum_{i=x}^{\infty }e^{-r\left(i+1\right)}\sum_{y=j}^{\beta }{s^{\prime }}_{iy}f_{iy}$$

Variances and standard errors (SE) for all parameters were calculated through 100,000 bootstrap replications [36,37]. 

The paired bootstrap test (a 5% significance level based on the confidence interval of difference) was applied to estimate the differences between the developmental and demographic parameters of the susceptible and all the resistant cohorts of *R. padi* [38].

### 2.6. Population Projection

The TIMING-MSChart program [39] was used to build population projections for both the susceptible and bifenthrin-, flonicamid-, and thiamethoxam-resistant cohorts following the methodology based on life table parameters [40]. The population of *R. padi* was projected continuously for a span of 50 days, assuming a scenario without the suppression of both biotic and abiotic factors. Each projected cohort initiated with 10 nymphs. Using a bootstrap approach, 100,000 bootstrap results of the finite rate of increase (*λ*) were sorted with subsequent organization of the outcomes for computation of the 2.5 and 97.5 percentiles, which resulted in the 2500th and 97,500th samples from the sorted bootstrap data. The life table datasets generated through the bootstrap method, corresponding to the 2.5 and 97.5 percentiles of *λ*, were then employed to project the population dynamics for again 50 days to elucidate the variation and uncertainty of the projected population depicted through the representation of confidence intervals [41].

## 3. Results

### 3.1. Selection-Induced Insecticide Resistance Development

Bifenthrin-, flonicamid-, and thiamethoxam-resistant strains of *R. padi* developed after being exposed to these insecticides repeatedly for ten generations under laboratory conditions were given at Table 1. The LC_50_ values of thiamethoxam, flonicamid, and bifenthrin of susceptible *R. padi* were 11.458, 5.710, and 18.863 ppm, respectively. Resistance development commenced gradually in the first three generations of the thiamethoxam-resistant strain (F1–F3), with LC_50_ values of 12.504, 17.656, and 21.052 ppm, respectively. However, a significant increase in the resistant ratio was observed from F4 to F10 generation with the LC_50_ values ranging from 39.996 to 394.846 ppm.

After ten generations of selection against thiamethoxam, *R. padi* evolved a 34.46-fold resistance. In contrast, the resistance ratio of the bifenthrin-induced strain increased from the F1 to F5 generations, with the LC_50_ values ranging from 24.621 to 99.397 ppm. However, the resistant development of *R. padi* increased dramatically from the F6 to F10 generation, with LC_50_ values of 141.104 to 603.210 ppm. Consequently, a 31.97-fold resistance was found after ten generations of selection against bifenthrin. On the other hand, flonicamid resistance increased gradually across all generations. The LC_50_ values spanned from 5.956 to 151.141 ppm, from the F1 to the F10 generations. *R. padi* developed a 26.46-fold resistance after being selected for flonicamid over ten generations (Table 1). 

### 3.2. Impact of Insecticide Resistance on Different Developmental Stages of R. padi

Life-history traits, including developmental time and longevity among bifenthrin-, flonicamid-, and thiamethoxam-resistant and susceptible strains of *R. padi,* are summarized in Table 2. The mean developmental durations of the first, third, and fourth instars aphids of thiamethoxam- and bifenthrin-resistant strains were significantly prolonged compared to flonicamid and the susceptible strain of *R. padi.* However, the second instar developmental duration did not exhibit a significant difference among all the resistant and susceptible strains of *R. padi*. Furthermore, the pre-adult period of *R. padi* was significantly increased in resistant strains of thiamethoxam, followed by bifenthrin, when compared to the flonicamid and susceptible strain. Conversely, the adult and total adult longevity was remarkably reduced in thiamethoxam, followed by bifenthrin and flonicamid in comparison to the susceptible aphids (Table 2).

### 3.3. Reproduction and Life Table Parameters of the Insecticide-Resistant Strain of R. padi

The comparison of the fecundity and life table parameters of susceptible and resistant strains of *R. padi* against bifenthrin, flonicamid, and thiamethoxam insecticides are presented in Table 3. The results revealed that the net reproductive rate (*R*_0_) of the thiamethoxam-, bifenthrin-, and flonicamid-resistant strains was significantly decreased compared to the susceptible strain. The fecundity (*F*) and the reproductive days (*RP_d_*) of the thiamethoxam-resistant strain were substantially decreased, followed by flonicamid and bifenthrin, compared to the susceptible strain. In addition, the intrinsic rate of increase (*r*) and the finite rate of increase (*λ*) were significantly lower in the resistant strains of thiamethoxam and bifenthrin when compared to the susceptible group of *R. padi*. However, no significant differences were observed in the flonicamid resistant population of *R. padi*. The mean generation time (*T*) value was significantly longer in bifenthrin and thiamethoxam-resistant strains compared to the flonicamid-resistant strain and susceptible *R. padi*. The total pre-reproductive period (TPRP) was significantly extended in thiamethoxam and bifenthrin when compared to susceptible aphids. Unlike the susceptible group of *R. padi*, no significant differences were observed in the adult pre-reproductive period (APRP) of the resistant aphids.

**Figure 1:**
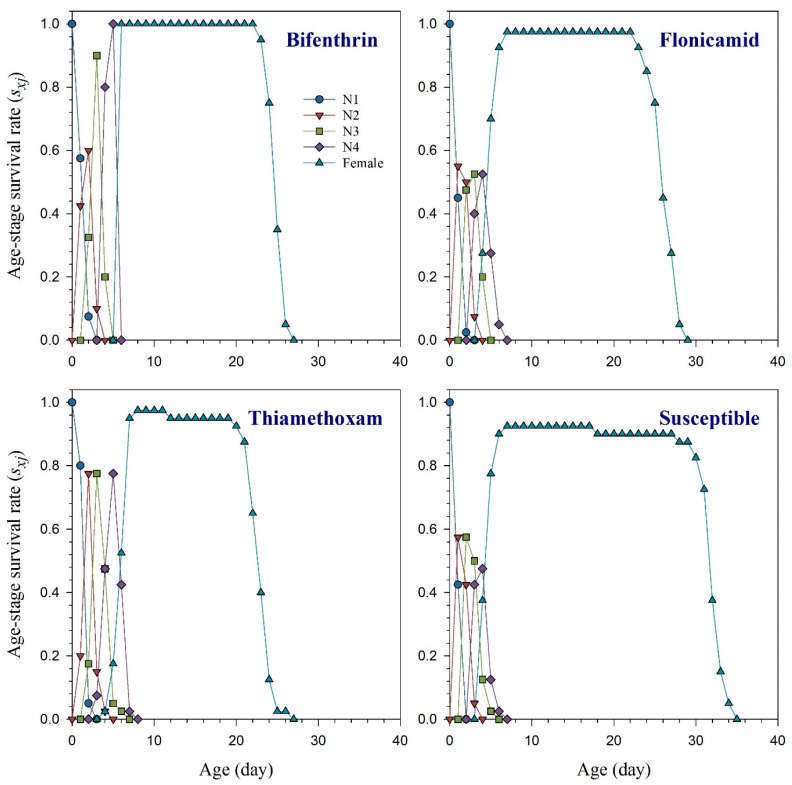
Age-stage specific survival rate (*s_xj_*) of bifenthrin-, flonicamid-, and thiamethoxam-resistant and susceptible strains of *Rhopalosiphum padi* [The figure uses different symbols to represent the developmental stages of *R. padi* as follows: circle for first-instar nymph (N1), triangle down for second-instar nymph (N2), square for third-instar nymph (N3), diamond for fourth-instar nymph, and triangle up for adult female].

**Figure 2:**
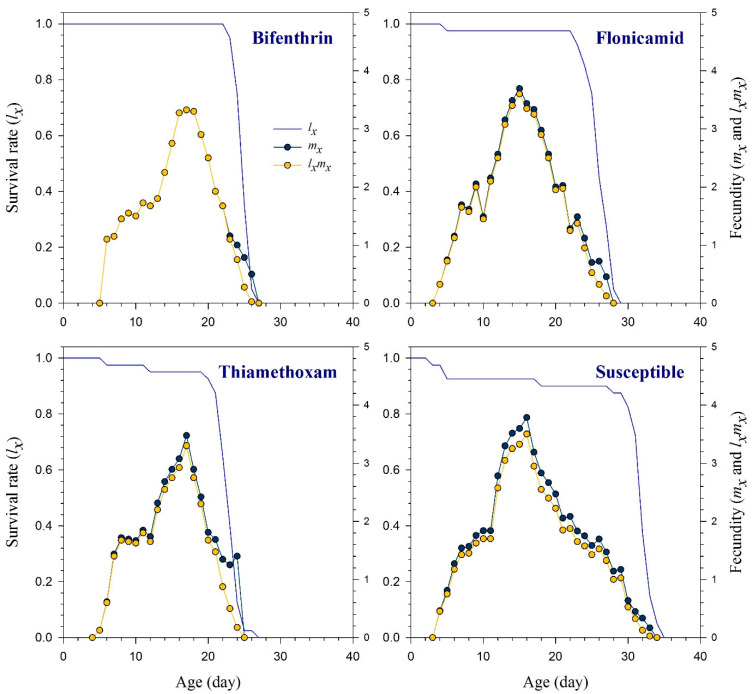
Age-specific survival rate (*l_x_*), age-specific fecundity (*m_x_*), and the age-specific maternity (*l_x_m_x_*) of bifenthrin-, flonicamid-, and thiamethoxam-resistant and susceptible strains of *Rhopalosiphum padi*.

**Figure 3:**
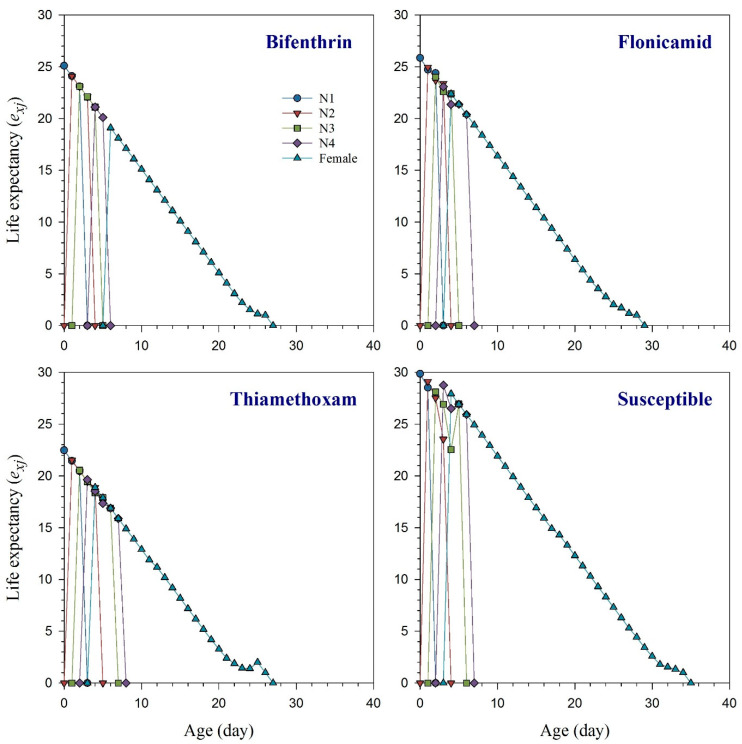
Age-stage life expectancy (*e_xj_*) of bifenthrin-, flonicamid-, and thiamethoxam-resistant and susceptible strains of *Rhopalosiphum padi* [The figure uses different symbols to represent the developmental stages of *R. padi* as follows: circle for first-instar nymph (N1), triangle down for second-instar nymph (N2), square for third-instar nymph (N3), diamond for fourth-instar nymph, and triangle up for adult female].

**Figure 4:**
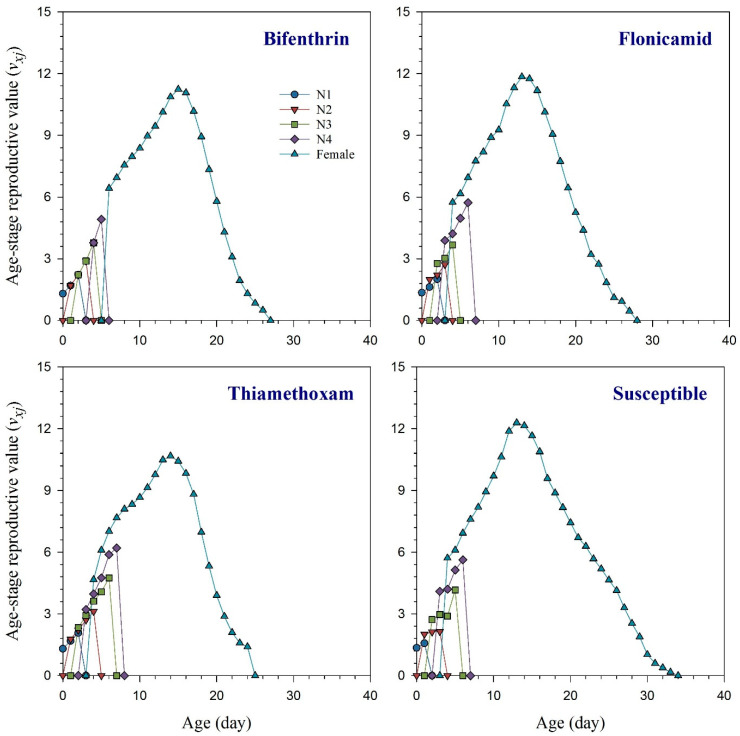
Age-stage reproductive value (*v_xj_*) of bifenthrin-, flonicamid-, and thiamethoxam-resistant and susceptible strains of *Rhopalosiphum padi* [The figure uses different symbols to represent the developmental stages of *R. padi* as follows: circle for first-instar nymph (N1), triangle down for second-instar nymph (N2), square for third-instar nymph (N3), diamond for fourth-instar nymph, and triangle up for adult female].

The age-stage specific survival rate (*s_xj_*) curves indicate the probability that a new-born *R. padi* nymph will survive to age *x* and stage *j* (Figure 1). These curves demonstrated that bifenthrin, flonicamid, and thiamethoxam had an adverse effect on the adult stage of *R. padi* compared to the susceptible strain. The effects of bifenthrin, flonicamid, and thiamethoxam resistance on the curves of age-specific survival rate (*l_x_*), age-specific fecundity (*m_x_*), and the age-specific maternity (*l_x_m_x_*) are illustrated in Figure 2 together with the susceptible group. Resistant aphids exhibited variations in their *l_x_*, *m_x_*, and *l_x_m_x_* curves compared to the susceptible strain of *R. padi*. Age-stage life expectancy (*e_xj_*) curves show how long a particular *R. padi* individual at age *x* and stage *j* is likely to survive after reaching age *x* (Figure 3). The *R. padi* resistant strain of bifenthrin, flonicamid, and thiamethoxam likely have shorter lifespans compared to susceptible aphids. Age-stage reproductive value (*v_xj_*) indicates the estimation of an age *x* and stage *j* population for their future progeny (Figure 4). The fecundity of *R. padi* in bifenthrin, flonicamid, and thiamethoxam significantly decreased compared to the susceptible population. 

### 3.4. Population Projection

**Figure 5:**
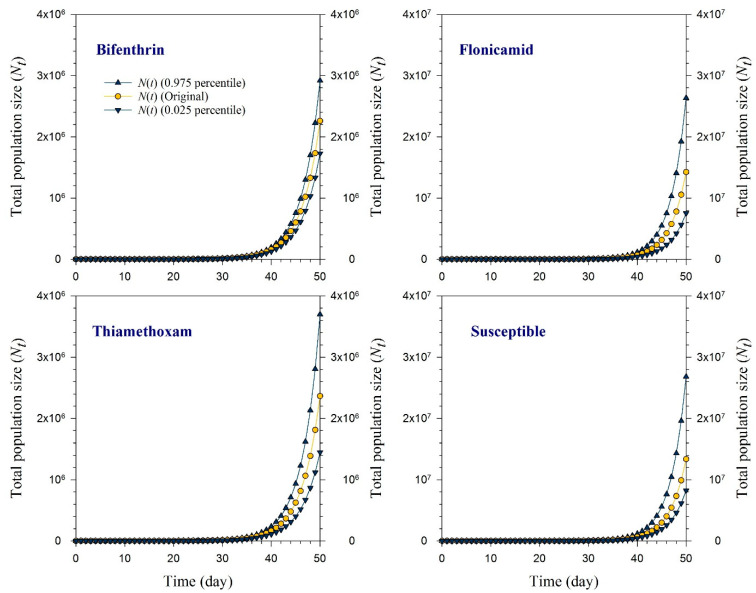
Projected total population size (*N_t_*) after 50 days for the bifenthrin-, flonicamid-, and thiamethoxam-resistant and susceptible strains of *Rhopalosiphum padi* utilizing life table data from the original cohort and the cohorts formed by incorporating the 2.5 and 97.5 percentiles of the finite rate of increase (*λ*).

The projected growth levels of susceptible and resistant strains of *R. padi* populations, including bifenthrin, flonicamid, and thiamethoxam resistance are plotted in Figure 5. In particular, strains originating from bifenthrin- and thiamethoxam-resistant populations displayed comparatively lower population sizes, reaching approximately 2,250,000 and 2,350,000 individuals, respectively. In contrast, the flonicamid-resistant strain and the susceptible strain of *R. padi* showed higher outcomes. The total population size peaked in the flonicamid-resistant strain, with a projection that exceeded 14,250,000 individuals after a 50-day period, while the susceptible *R. padi* strain yielded an estimated population size of almost 13,350,000 individuals by the end of the 50-day projection. 

## 4. Discussion

Chemical insecticides are extensively used for controlling aphids throughout the world. However, the emergence of insecticide resistance considerably reduced the effectiveness of insecticides [10,11,24]. Various insect pests, such as *A. gossypii, M. persicae*, *Bemisia tabaci* (Gennadius) (Hemiptera: Aleyroridae), *Leptinotarsa decemlineata* (Coleoptera: Chrysomelidae), *Bradysia odoriphaga* Yang et Zhang (Diptera: Sciaridae), *N. lugens*, *Frankliniella occidentalis* (Pergande) (Thysanoptera: Thripidae) and *Heliothis virescens* (F.) (Lepidoptera: Noctuidae), have been subject of several experiments aimed to develop insecticide resistance [10,11,15,42,43,44,45]. The evolution of resistance associated with fitness costs significantly influence pesticide resistance [17]. No previous research has previously investigated the fitness costs associated with selection-induced bifenthrin, flonicamid, and thiamethoxam resistance in the *R. padi*. Consequently, it is crucial to assess the potential risk of resistance development and associated fitness cost in *R. padi* to inform effective resistance management strategies.

The results indicated that *R. padi* developed resistance levels of 34.46-fold to thiamethoxam, 31.97-fold to bifenthrin, and 26.46-fold to flonicamid after ten generations of selection under laboratory conditions. In a recent study, 43.32-fold resistance of chlorfenapyr was reported in *B. odoriphaga* after laboratory selection for ten generations [46]. Similarly, a 76-fold clothianidin resistance was found in *B. odoriphaga* following ten generations of selections [10]. In other cases, *Spodoptera exigua* (Hübner) (Lepidoptera: Noctuidae) exhibited a 69.76-fold resistance to deltamethrin and 113.29-fold gossypol resistance again after ten generations of laboratory selection [47]. These findings highlighted the occurrence of resistance evolution in different insect pests, which were subjected to ten generations of selection pressure. Several other studies have reported selection-induced insecticide resistance in different insects against insecticides [27,48,49,50,51]. Differences in resistance fold values may be attributed to variations in the initial sensitivity of field-collected populations before insecticide treatment and the duration of rearing the colony under insecticide-free laboratory conditions. It is conceivable that *R. padi* could develop even higher levels of resistance to thiamethoxam, bifenthrin, and flonicamid with extended selection pressure over generations.

The resistance-induced fitness costs have been thoroughly investigated in various insect pests, including *N. lugens*, *B. odoriphaga*, *M. domestica*, *T. hawaiiensis*, and *P. xylostella* [10,11,19,20,21,22]. In this study, we selected four strains of *R. padi* with a similar genetic background to accurately quantify resistance-related fitness costs. Our results showed significant differences in the developmental stages of *R. padi* among thiamethoxam and bifenthrin resistant strains when compared to the susceptible population. However, no significant differences were found on the developmental duration of the second instar between the susceptible and resistant strains. Specifically, the pre-adult period was considerably prolonged in the thiamethoxam-resistant strain, followed by bifenthrin, compared to flonicamid and the susceptible strain. These findings align with a previous study that imidacloprid-resistant *A. gossypii* considerably extended the developmental and pre-adult stages compared to susceptible strain [52]. Similarly, the developmental stages of the resistant strain of *N. lugens* have also shown significant extension [22]. A prolonged developmental period was noted in thiamethoxam-resistant *A. gossypii* strains [53]. Additionally, the resistant strain has improved developmental phases of *B. odoriphaga* against clothianidin [10]. Further investigation has shown that strains of *M. domestica, H. virescens*, and *Helicoverpa armigera* (Hübner) (Lepidoptera: Noctuidae) that were resistant to imidacloprid, indoxacarb, and deltamethrin exhibited longer pre-adult developmental durations [15,54,55]. These findings demonstrated that extended developmental stages in resistant aphids are one of the primary fitness costs associated with insecticide resistance. It suggests that without insecticide selection pressure, resistant strains of *R. padi* to thiamethoxam, bifenthrin, and flonicamid would not spread quickly in the field. On the contrary, some studies have also reported a decline in the developmental duration of pre-adult stages of insect pests [23,24,56].

Bifenthrin-, flonicamid-, and thiamethoxam-resistant strains of *R. padi* had significantly reduced fecundity, adult longevity, and reproductive days compared with the susceptible strain. These findings support our previous research, which demonstrated that susceptible *A. gossypii* strains had much longer reproductive days, lifespans, and fecundities than resistant strains [23,56]. Thiamethoxam-resistant *A. gossypii* also exhibited reduced fecundity, longevity, and reproductive days [53]. Additionally, the decreased longevity and fecundity were also observed in the resistant strains of *B. odoriphaga*, *M. persicae*, and *S. exigua* [10,25,47]. Demographic traits provide insights into the growth potential of the insect population. In our study, key demographic parameters such as the net reproductive rate (*R*_0_), the intrinsic rate of increase (*r*), and the finite rate of increase (*λ*) were significantly reduced in bifenthrin-, flonicamid-, and thiamethoxam-resistant strains as compared to susceptible aphids. Similar results were reported by [53], where the intrinsic rate of increase (*r*), the finite rate of increase (*λ*), and the net reproductive rate (*R*_0_) were substantially decreased in thiamethoxam-resistant strains of *A. gossypii* [53]. These results indicated that the selection pressure of insecticides (bifenthrin, flonicamid, and thiamethoxam) affected the growth potential of *R. padi*.

The trade-off among life history parameters occurs when they are energetically costly and insects need energy and resources for adaptation and survival under stress conditions of insecticide toxicity [17]. A previous study investigated how imidacloprid resistance in the *N. lugens* affects life history traits and energy reserves [57]. Imidacloprid-resistant female insects had shorter survival and lower fecundity compared to susceptible strains, resulting in lower net reproductive rates (*R*_0_), but they stored more lipids, indicating a physiological trade-off where energy reserves were used for metabolic detoxification rather than reproduction [57]. Likewise, the widespread development of insecticide resistance in vector populations is often associated with fitness costs, including reduced preimaginal survival, adult size, longevity, and fecundity. These pleiotropic effects are commonly explained by resource-based trade-offs, where insecticide resistance depletes energetic stores. In a study involving *Culex pipiens* L. (Diptera: Culicidae) resistant to insecticides through esterase overproduction and acetylcholinesterase modification and these mechanisms led to reduced energetic reserves, supporting the trade-off theory. However, in acetylcholinesterase-modified mosquitoes, resource depletion may result from hyperactivation of the nervous system rather than resource-based trade-offs [58]. 

The selection pressure of bifenthrin, flonicamid and thiamethoxam may be responsible for the reduction in longevity and fecundity of resistant strains, indicating the energy trade-off between adaptation and other attributes, e.g., longevity and fecundity as stated by the life-history theory. Over the past ten years, fitness costs have been thoroughly examined, and several studies have shown fitness cost scenarios in insect species that developed resistance to various insecticides [10,11,17]. In general, this study reported that *R. padi* has the potential to develop resistance against bifenthrin, flonicamid, and thiamethoxam under continuous selection pressure. Furthermore, our current study clearly underlined the resistance-induced fitness costs in all resistant strains indicating the direct impact of insecticide resistance on the growth potential of resistant insects. 

## 5. Conclusions

Overall, this study demonstrated the evolution of bifenthrin, flonicamid, and thiamethoxam resistance in *R. padi* following ten consecutive generations of selection under laboratory conditions. Furthermore, we have reported resistance-induced fitness costs such as reduced longevity, fecundity, and key demographic traits in bifenthrin-, flonicamid-, and thiamethoxam-resistant strains compared to susceptible strains. The current study provides important information about developing resistance to these commonly used insecticides and the associated fitness costs in *R. padi*. These results might provide in-depth details about the potential risk of widely used insecticides and their overall impact on the target insects that will ultimately help resistance management strategies against key insect pests. However, the current study was conducted under laboratory conditions which may not translate well into population effects under field conditions. Therefore, future studies are required under field contexts, which are necessary to understand the evolution of insecticide resistance and their impact on the overall fitness of insect pests.

## Figures and Tables

**Table 1:** Laboratory-induced selection of *Rhopalosiphum padi* for resistance against thiamethoxam, flonicamid, and bifenthrin.

Generations	Insecticides	LC_50_ (95%CI) ^a^ ppm	Slope ± SE ^b^	*χ* ^2^	*p*-Value	RR ^c^
F0	Thiamethoxam	11.458 (9.766–13.416)	2.718 ± 0.283	5.458	0.964	-
	Flonicamid	5.710 (4.869–6.746)	2.453 ± 0.238	6.147	0.940	-
	Bifenthrin	18.863 (16.230–21.958)	2.719 ± 0.256	11.039	0.607	-
F1	Thiamethoxam	12.504 (10.608–14.755)	2.581 ± 0.275	4.914	0.977	1.09
	Flonicamid	5.956 (5.072–7.039)	2.442 ± 0.239	3.393	0.996	1.04
	Bifenthrin	24.621 (21.057–28.616)	2.987 ± 0.341	9.519	0.732	1.31
F2	Thiamethoxam	17.656 (14.602–22.031)	2.030 ± 0.224	3.735	0.994	1.54
	Flonicamid	6.800 (5.594–8.428)	1.882 ± 0.209	3.366	0.996	1.19
	Bifenthrin	36.702 (31.064–44.364)	2.433 ± 0.255	6.840	0.910	1.94
F3	Thiamethoxam	21.052 (17.013–27.463)	1.852 ± 0.220	3.846	0.993	1.83
	Flonicamid	12.232 (9.572–17.022)	1.691 ± 0.236	3.206	0.997	2.14
	Bifenthrin	53.151 (43.078–69.989)	2.063 ± 0.253	3.854	0.992	2.81
F4	Thiamethoxam	39.996 (31.959–53.067)	1.716 ± 0.211	8.047	0.841	3.49
	Flonicamid	19.423 (15.319–26.205)	1.623 ± 0.217	4.248	0.988	3.40
	Bifenthrin	73.771 (56.240–110.790)	1.822 ± 0.258	6.710	0.916	3.91
F5	Thiamethoxam	60.509 (50.436–74.271)	2.088 ± 0.223	12.067	0.522	5.28
	Flonicamid	35.908 (30.070–43.689)	2.367 ± 0.289	4.520	0.984	6.28
	Bifenthrin	99.397 (79.869–132.243)	1.920 ± 0.235	7.830	0.854	5.26
F6	Thiamethoxam	102.398 (86.485–122.681)	2.271 ± 0.231	12.708	0.470	8.93
	Flonicamid	47.424 (40.097–56.463)	2.298 ± 0.229	3.436	0.995	8.31
	Bifenthrin	141.104 (110.687–200.683)	2.039 ± 0.279	4.320	0.987	7.48
F7	Thiamethoxam	170.830 (139.511–219.495)	1.986 ± 0.230	7.188	0.892	14.90
	Flonicamid	73.741 (59.665–94.896)	1.833 ± 0.228	4.652	0.982	12.91
	Bifenthrin	219.672 (179.875–284.911)	2.294 ± 0.304	7.717	0.861	11.64
F8	Thiamethoxam	258.040 (207.098–350.288)	2.176 ± 0.284	4.432	0.985	22.52
	Flonicamid	84.169 (69.397–106.080)	2.167 ± 0.275	3.114	0.997	14.74
	Bifenthrin	326.191 (244.979–508.106)	1.838 ± 0.268	6.442	0.928	17.29
F9	Thiamethoxam	335.723 (287.430–391.916)	3.385 ± 0.498	4.913	0.977	29.30
	Flonicamid	109.285 (93.127–128.223)	2.754 ± 0.305	3.814	0.993	19.13
	Bifenthrin	442.158 (352.672–599.054)	1.939 ± 0.243	7.605	0.868	23.44
F10	Thiamethoxam	394.846 (324.190–506.346)	2.171 ± 0.253	7.709	0.862	34.46
	Flonicamid	151.141 (120.502–200.341)	1.659 ± 0.207	3.145	0.997	26.46
	Bifenthrin	603.210 (480.636–842.423)	2.315 ± 0.326	3.398	0.996	31.97

Exposed larvae in bioassay, including control = 360; df = 13. ^a^ 95% confidence intervals. ^b^ Standard error. ^c^ RR, resistance ratio, analyzed as (LC_50_ of resistant strain/LC_50_ of susceptible strain).

**Table 2:** Duration (days) different developmental stages (mean ± SE) of resistant (bifenthrin, flonicamid, and thiamethoxam) and susceptible strain of *Rhopalosiphum padi*.

Stage		Susceptible		Bifenthrin		Flonicamid		Thiamethoxam
	*n*	Mean ± SE	*n*	Mean ± SE	*n*	Mean ± SE	*n*	Mean ± SE
First-instar nymph	40	1.43 ± 0.08 b	40	1.65 ± 0.10 ab	40	1.48 ± 0.09 b	40	1.85 ± 0.08 a
Second-instar nymph	40	1.05 ± 0.03 a	40	1.13 ± 0.05 a	40	1.13 ± 0.05 a	40	1.15 ± 0.06 a
Third-instar nymph	38	1.21 ± 0.07 b	40	1.43 ± 0.08 a	40	1.20 ± 0.06 b	40	1.50 ± 0.08 a
Fourth-instar nymph	37	1.11 ± 0.05 b	40	1.80 ± 0.06 a	39	1.26 ± 0.07 b	39	1.77 ± 0.08 a
Total pre-adult stages	37	4.78 ± 0.13 c	40	6.01 ± 0.01 b	39	5.05 ± 0.14 c	39	6.28 ± 0.14 a
Adult longevity	37	27.14 ± 0.44 a	40	19.10 ± 0.15 c	39	21.33 ± 0.17 b	39	16.62 ± 0.34 d
Total longevity	37	31.92 ± 0.44 a	40	25.10 ± 0.15 c	39	26.38 ± 0.24 b	39	22.90 ± 0.35 d

Standard errors were estimated using the bootstrap technique with 100,000 resamples. The difference was compared using the paired bootstrap test (*p* < 0.05). The means within a row followed by a different lowercase letters indicate significant differences among the strains.

**Table 3:** Reproduction and life table parameters (mean ± SE) of resistant (bifenthrin, flonicamid, and thiamethoxam) and susceptible strain of *Rhopalosiphum padi*.

Parameters ^a^	Susceptible	Bifenthrin	Flonicamid	Thiamethoxam
	Mean ± SE	Mean ± SE	Mean ± SE	Mean ± SE
*R*_0_ (offspring/individual)	50.35 ± 2.65 a	38.00 ± 0.65 c	44.00 ± 1.42 b	34.00 ± 1.28 d
*r* (day^−1^)	0.3008 ± 0.0069 a	0.2657 ± 0.0027 b	0.3021 ± 0.0062 a	0.2667 ± 0.0047 b
*λ* (day^−1^)	1.3510 ± 0.0093 a	1.3044 ± 0.0035 b	1.3527 ± 0.0084 a	1.3056 ± 0.0062 b
*T* (days)	13.03 ± 0.21 bc	13.69 ± 0.11 a	12.53 ± 0.22 c	13.22 ± 0.19 b
*F* (nymphs/female)	54.43 ± 1.50 a	38.00 ± 0.65 c	45.13 ± 0.91 b	34.87 ± 0.96 d
*RP_d_* (days)	24.22 ± 0.59 a	18.00 ± 0.17 c	19.28 ± 0.23 b	15.69 ± 0.35 d
APRP (days)	0.35 ± 0.11 a	0.20 ± 0.08 a	0.41 ± 0.11 a	0.28 ± 0.08 a
TPRP (days)	5.14 ± 0.17 c	6.20 ± 0.08 b	5.46 ± 0.19 c	6.56 ± 0.12 a

Standard errors were estimated using the bootstrap technique with 100,000 resamples. The difference was compared using the paired bootstrap test (*p* < 0.05). The means within a row followed by a different lowercase letters indicate significant differences among the strains. ^a^ *R*_0_ = net reproductive rate; *r =* intrinsic rate of increase; *λ* = finite rate of increase; *T* = mean generation time; *F* = fecundity; *RP_d_* = reproductive days; APRP = adult pre-reproductive period; TPRP = total pre-reproductive period.

## Data Availability

All data analyzed during this study are included in this published article.
